# Work status changes and associated factors in a nationwide sample of Norwegian long-term breast cancer survivors

**DOI:** 10.1007/s11764-022-01202-2

**Published:** 2022-03-22

**Authors:** Synne-Kristin Hoffart Bøhn, K. F. Vandraas, C. E. Kiserud, A. A. Dahl, L. Thorsen, M. Ewertz, H. C. Lie, R. Falk, K. V. Reinertsen

**Affiliations:** 1 National Advisory Unit for Late Effects After Cancer Treatment, Department of Oncology, Oslo University Hospital, Radiumhospitalet, Oslo Norway; 2 Faculty of Medicine, University of Oslo, Oslo, Norway; 3 Division of Cancer Medicine, Department of Clinical Service, Oslo University Hospital, Oslo, Norway; 4 Oncology Research Unit, Institute of Clinical Research, University of Southern Denmark, Odense, Denmark; 5 Faculty of Medicine, Department of Behavioural Medicine, Institute of Basic Medical Sciences, University of Oslo, Oslo, Norway; 6 Oslo Centre for Biostatistics and Epidemiology, Research Support Services, Oslo University Hospital, Oslo, Norway

**Keywords:** Breast cancer, Return to work, Work sustainability, Survivorship, Late effects

## Abstract

**Purpose:**

The study aims to describe work status at diagnosis and 8 years post-diagnosis in a nationwide sample of breast cancer survivors (BCSs), and investigate associated and self-reported factors of reduced work status.

**Methods:**

Women aged 20–65 years when diagnosed with stage I–III breast cancer (BC) in 2011 or 2012 were invited to participate in a questionnaire study in 2019 (*n* = 2803), of whom 49% (*n* = 1361) responded. For this sub-study, we included 974 BCSs below the legal retirement age in Norway (< 67 years) at survey and with complete work status data. Reduced work status was defined as being in paid work at BC diagnosis and not working at time of survey. Logistic regression analyses were applied to identify factors associated with reduced work status.

**Results:**

Of BCSs who were in paid work at diagnosis (*n* = 845), 63% maintained their work status to 8 years later. Reduced work status was associated with not living with children (OR .44, 95% CI .24–.82), age (OR 1.16, 95% CI 1.11–1.21), chemotherapy (OR 2.83, 95% CI 1.24–6.61), > 2 comorbid conditions (OR 2.27, 95% CI 1.16–4.32), cognitive function (OR .99, 95% CI .98–.99), fatigue (OR 1.02, 95% CI 1.01–1.03), and neuroticism (OR 1.57, 95% CI 1.00–2.46). BC and late effects were reported as reasons for reduced work status and disability.

**Conclusions:**

The majority of BCSs who were in paid work at diagnosis were working 8 years later.

**Implications for Cancer Survivors:**

Our results suggest a need to focus on fatigue and reduced cognitive function among long-term BCSs, with the ultimate aim of improving work sustainability.

**Supplementary Information:**

The online version contains supplementary material available at 10.1007/s11764-022-01202-2.

## Background

Each year, around 3700 women are diagnosed with breast cancer (BC) in Norway, of whom approximately 60% are within working age, i.e., 18–67 years old [1]. Due to early detection and effective treatments, the 5-year relative survival ranges from 80 to 100% for stage I–III BC in Norway and other Western countries [1, 2]. Thus, the number of long-term breast cancer survivors (BCSs) within working age is increasing.

Considering their relatively low age at diagnosis and long lifetime expectancy, work impairments among long-term BCSs pose substantial costs at a personal, familial, and societal level. Being part of the work force after BC is important, not only for income, but also for self-esteem, use of personal resources, and social status, and may help BCSs to regain normality after their malignancy [3].

Patients with early-stage BC often receive highly intensive treatments, including combinations of surgery, radiotherapy, and systemic therapies, and have a high risk of late effects [4]. Common late effects in BCSs include fatigue, pain, neuropathy, lymphedema, mental distress, fear of recurrence, and cognitive impairments [4], which may have substantial negative consequences for physical and psychosocial functioning, including reduced work participation [5–8].

Research on work outcomes in BCSs is mainly limited to the early post-BC treatment period, showing that the majority return to work within the first 2 years post-diagnosis [5, 9–11]. Factors restricting return to work among early BCSs comprise socio-demographic and work-contextual issues, personal preferences, and mental and somatic health, including treatment and adverse effects [5, 9, 10]. However, the employment rate may change after this point, e.g., due to late effects that persist or appear several months or even years after treatment. Three cross-sectional studies from various countries have described that > 60% of BCSs are employed approximately 5 years from diagnosis [12–14]. On the other hand, two register-based studies comparing long-term BC survivors to normative samples have reported lower employment rates in BCSs [15, 16], indicating that BCSs have challenges maintaining work status also beyond the first 5 years post-diagnosis. Women in these studies were diagnosed with BC during 1992–2005, and as patient-reported outcome measures were not included as explanatory variables [15, 16] self-perceived reasons for reduced work status or receipt of disability pension among long-term BCSs was not assessed. Thus, knowledge is scarce and conflicting on work status changes and patient-reported associated factors among long-term BCSs treated more recently.

Identifying individual and modifiable characteristics of BCSs with reduced work status may guide rehabilitation programs and clinical efforts to improve work sustainability. Multidisciplinary interventions including regular physical exercise have shown to enhance return to work after cancer [17], and are positively associated with return to work up to 3 years after BC diagnosis [18]. The association between physical activity and work participation in long-term BCSs has received little attention. Other factors such as the personality trait neuroticism and health literacy impact on BCSs health, lifestyle, and quality of life [19, 20], but their relation to work participation among long-term BCSs have scarcely been investigated.

The aims of this population-based cross-sectional study of long-term Norwegian BCSs are to (1) explore work status at diagnosis and at 8 years post-diagnosis for BCSs within working age, (2) investigate factors associated with reduced work status from diagnosis to survey among BCSs who were in paid work at diagnosis, and (3) examine self-perceived reasons for reduced work status or disability pension. The analyses in the present study are based on the Cancer and Work model developed by Feuerstein et al. [17], including variables related to health, symptoms, and function.

## Material and methods

### Study sample

The study sample was extracted from the Norwegian SWEET-study (**S**urvivorship-Work-sExual hEalTh-study), a cross-sectional, questionnaire-based study addressing work and sexual health among females diagnosed with stage I–III BC in 2011 or 2012 at the age of 20–65 years. For the present sub-study, we included BCSs below the legal retirement age in Norway (67 years) at survey. BCSs were identified by the Cancer Registry of Norway (CRN), and they had to be free of BC recurrence or second malignancies, except for non-melanoma skin cancer and ductal carcinoma in situ. Invitations were mailed to 2803 BCSs during fall 2019. Non-responders received one reminder during spring 2020.

A total of 1361 BCSs responded (49%). We excluded BCSs with incomplete consent (*n* = 3), self-reported BC recurrence (*n* = 3), missing information on work status at diagnosis or survey (*n* = 31), those who had reached the legal retirement age in Norway of 67 years at survey (*n* = 349), and one participant that had retired early at diagnosis. Thus, data from 974 BCSs were included in the analysis.

### Main outcome variables

The main outcomes were self-reported work status and changes in work status from BC diagnosis to survey.

BCSs reported their work status at diagnosis and at survey by the following alternatives: full time work, part time work, self-employed, sick leave, disability pension, retired, job seeker, temporarily laid off, work allowance, education or military service, homemaker, and/or other status. These alternatives were categorized as paid work (working fulltime, part-time, being self-employed, or on sick leave), disability pension, retired, and other work statuses (the remaining alternatives).

Changes in work status from diagnosis to survey were further explored among the 845 BCSs who were in paid work at diagnosis. Change in work status was dichotomized into “reduced work status” (transition from being in paid work at diagnosis to not holding paid work at survey) versus “maintained work status” (still in paid work at survey).

Among those with reduced work status, those not working were asked to respond to whether late effects after BC were the reason for not working (“yes, partly”/”yes, mainly”/ “no”), while those receiving disability pension were asked if BC had caused work disability (“yes”/ “no”/ “do not know”).

### Explanatory variables

#### Variables extracted from the Cancer Registry of Norway

Age at diagnosis, BC stage, and surgical treatment were retrieved from the CRN.

#### Self-reported variables

Treatment information was categorized as no systemic treatment, chemotherapy only, endocrine therapy only, chemotherapy and endocrine therapy, chemotherapy and trastuzumab, and chemotherapy, trastuzumab, and endocrine therapy.

Socio-demographic variables included living status (with partner or children ≤ 18 years), and education (long, > 12 years versus short, ≤ 12 years).

Somatic comorbid conditions were assessed by history of the following: cardiovascular, kidney, thyroid, gastrointestinal, rheumatic, and pulmonary diseases; diabetes; arthrosis; muscle/joint pain; and epilepsy. Number of comorbidities were categorized into no/1–2/ > 2 comorbid conditions.

Sleep problems were defined as more than three episodes per week of difficulty falling asleep and/or waking up too early without being able to go back to sleep, over the past 3 months, using two items from the Nord-Trøndelag Health Study [21]. Responses were dichotomized into “yes” (often or almost every night) and “no” (never or occasionally). 

Neuropathy was assessed using two items from the scale for chemotherapy induced long-term neurotoxicity (SCIN) [22]. Presence of peripheral sensory neuropathy in hands and feet were rated from 0 (not at all) to 3 (very much), providing a sum score from 0 to 6. The sum score was dichotomized into high (≥ 4) and low (≤ 3) degree of neuropathy.

Cognitive function, pain, and fatigue were reported using the European Organization for Research and Treatment of Cancer Quality of Life Questionnaire (EORTC QLQ-C30), version 3, while arm and breast symptoms were assessed by the EORTC QLQ breast cancer specific module (EORTC BR23) [23, 24]. The EORTC scoring algorithms were used [23], and items were rated from 1 (not at all) to 4 (very much), and then transformed to a 0 to 100 scale. High cognitive functioning yields a high functional score, while more severe pain, fatigue, arm, and/or breast symptoms result in higher symptom scores. In this study, Cronbach’s alphas were 0.72 (cognitive functioning), 0.87 (pain), 0.89 (fatigue), 0.79 (arm symptoms), and 0.78 (breast symptoms).

Depressive symptoms were assessed by the nine-item Patient Health Questionnaire (PHQ-9) [25]. The PHQ-9 assesses the frequency of depressive symptoms during the last 2 weeks. Response categories range from 0 (not at all) to 3 (nearly every day), providing a sum score from 0 to 27, with higher scores reflecting higher level of depressive symptoms. Cronbach’s alpha was 0.80.

Anxiety symptoms were assessed by the General Anxiety Disorder 7-item scale (GAD-7) [26], which consists of seven items self-rating worry and anxiety symptoms during the last 2 weeks. Each item is scored from not at all (0) to nearly every day (3) with total scores ranging from 0 to 21. Higher scores reflect increasing anxiety levels. Cronbach’s alpha was 0.90.

Fear of Cancer Recurrence (FCR) was measured using four items from the Concern About Recurrence Questionnaire (CARQ) [27]. The first three items assess the frequency and degree of distress caused by FCR, scored from 0 (not at all) to 10 (a great deal). The fourth item asks respondents to quantify their perceived risk of recurrence as a number from 0 to 100%. Scores on item 4 was transformed to a score from 0 to 10, in consistency with the other three items. A total sum score ranging from 0 to 40 was calculated, with higher scores reflecting more FCR. Cronbach’s alpha for these four items from CARQ was 0.70.

The basic personality trait of neuroticism was assessed using six items from the abridged version of Eysenck Personality Inventory [28, 29]. Items were scored as present (1) or absent (0). A total score ranging from 0 to 6 was calculated and dichotomized into high (sum scores 3–6) and low neuroticism (sum scores 0–2) [30, 31]. Cronbach’s alpha for these six items from Eysenck Personality Inventory was 0.80.

Health literacy was measured by the European Health Literacy Survey Questionnaire-12 (HLS-Q12) [32]. HLS-Q12 assesses the ability to access, understand, appraise, and apply health information across three different health care settings. Response categories range from very difficult (1) to very easy (4), providing a sum score from 12 to 48. A higher score reflects higher levels of HL. Cronbach’s alpha was 0.90.

Obesity was classified as body mass index (BMI) ≥ 30 kg/m^2^, calculated from height and body weight [33]. Smoking was assessed by the question “Do you smoke daily?,” and categorized as yes versus no.

Physical activity was assessed by a modified version of the Godin Leisure Time Exercise Questionnaire (GLTEQ) [34, 35]. The GLTEQ assesses the average frequency and number of minutes of mild, moderate, and vigorous leisure-time physical activity during a typical week. The number of minutes within the different intensity levels were calculated for each participant, and used to classify individuals as physically active (≥ 150 min of moderate intensity or ≥ 75 min of vigorous intensity per week) or inactive according to guidelines [36].

### Statistical analyses

Continuous variables were described by mean and standard deviation (SD), and categorical variables as numbers and percentages. Missing values were handled according to guidelines or common practice for each of the questionnaires. For the GAD-7 and Eysenck Personality Inventory, missing values were substituted with mean imputation procedure when more than 50% of items had been answered. For the PHQ-9 and the HLS-Q12, mean imputation procedure was performed if no more than two items were missing. Otherwise, no imputations were performed.

Logistic regression analyses were applied to investigate the associations between explanatory variables and risk of reduced work status, estimating odds ratios (ORs) with 95% confidence intervals (95% CIs). Variables associated with reduced work status in univariable analyses with *p* < 0.25 were included in multivariable analyses. We excluded the anxiety variable due to conceptual overlap with fear of recurrence, but no other multicollinearity was observed (variation inflation factors < 1.5). The assumption of a linear relationship between the continuous variables and the log odds of reduced work status was fulfilled.

As a sensitivity analysis, we compared data from the CRN among responders and non-responders to the SWEET study. Further, socio-demographic and clinical characteristics among participating BCSs who were outside the work force at BC diagnosis were described.

An association with a *p*-value < 0.05 was considered statistically significant. All analyses were performed using IBM SPSS statistics version 26 (Armonk, NY).

## Results

At survey, 8 years after diagnosis, the mean age of the BCSs was 56 years (Table 1). More than half of the BCSs had long education. All had undergone surgery, 79% had also received radiotherapy, and 78% had received chemotherapy. Three of four BCSs reported at least one comorbid condition, 17% were obese and 56% did not meet the public physical activity guidelines (Table 1).

**Table 1:** Characteristics of long-term breast cancer survivors (BCSs) from Norway (*n* = 974), by change in work status

	Analysis sample	Change in work status from diagnosis to 8 years post-diagnosis among BCSs in paid work at diagnosis and at working age at survey		BCSs outside the workforce at diagnosis
	*n* = 974	Maintained *n* = 530	Reduced *n* = 315	*n* = 129
Socio-demographic variables				
Age at survey, mean (SD)	56.0 (7.0)	54.8 (6.4)	57.6 (7.2)	57.3 (7.6)
Living with partner, *n* (%)	741 (76)	411 (78)	233 (74)	97 (75)
Living with children < 18 years, *n* (%)	196 (20)	125 (24)	52 (17)	19 (15)
Years of education, *n* (%) (*n* = 969)				
Long (> 12 years)	548 (57)	321 (61)	177 (57)	50 (39)
Short (≤ 12 years)	420 (43)	207 (39)	135 (43)	78 (61)
Cancer-related variables				
Age at diagnosis, mean (SD)	48.1 (6.9)	46.9 (6.4)	49. 6 (7.2)	49.2 (7.6)
Years since diagnosis, mean (SD)	8.0 (0.7)	8.0 (0.7)	8.0 (0.7)	8.1 (0.7)
Stage, *n* (%)				
I	386 (40)	216 (41)	116 (37)	54 (48)
II	374 (38)	207 (39)	115 (37)	52 (40)
III	84 (9)	41 (8)	36 (11)	7 (5)
*Missing*	*130 (13)*	*66 (13)*	*48 (15)*	*16 (12)*
Treatment, *n* (%)				
BCT	530 (54)	280 (53)	177 (56)	73 (57)
Mastectomy	444 (46)	250 (47)	138 (44)	56 (43)
Radiotherapy	772 (79)	417 (79)	256 (81)	99 (77)
*Systemic treatment (n* = *967)*				
No systemic treatment	130 (13)	74 (14)	34 (11)	22 (17)
Chemotherapy alone	121 (13)	59 (11)	45 (14)	17 (13)
Endocrine treatment alone	79 (8)	32 (6)	31 (10)	16 (12)
Chemotherapy + endocrine therapy	445 (46)	251 (47)	138 (44)	56 (43)
Chemotherapy + trastuzumab	53 (6)	33 (6)	12 (4)	8 (6)
Chemotherapy + trastuzumab + endocrine therapy	138 (14)	76 (14)	52 (17)	10 (8)
Health variables, *n* (%)				
Somatic comorbid conditions (*n* = 970)				
0	239 (25)	176 (33)	49 (16)	14 (11)
1–2	540 (56)	291 (55)	183 (58)	65 (51)
> 2	192 (20)	62 (12)	82 (26)	48 (38)
Sleep problems^1^ (*n* = 964)	453 (47)	213 (40)	175 (56)	65 (52)
Neuropathy (*n* = 962)	204 (21)	76 (14)	95 (30)	33 (26)
High neuroticism (*n* = 959)	389 (41)	164 (31)	160 (51)	65 (50)
Health variables, mean (SD)				
*EORTC-QLQ-C30/BR-23 (score 0–100)*				
Cognitive function^2^ (*n* = 970)	71.4 (25.5)	77.1 (22.7)	64.4 (26.7)	64.8 (27.7)
Pain^3^ (*n* = 970)	28.7 (29.6)	20.1 (24.9)	35.9 (30.5)	46.6 (32.3)
Fatigue^3^ (*n* = 970)	40.8 (27.7)	32.7 (24.8)	49.6 (27.5)	53.0 (28.8)
Arm symptoms^3^ (*n* = 964)	22.3 (25.2)	18.3 (22.5)	27.2 (28.1)	26.8 (25.6)
Breast symptoms^3^ (*n* = 964)	17.0 (19.6)	14.0 (17.3)	19.7 (21.9)	22.6 (21.0)
Depressive symptoms^3^ (score 0–27) (*n* = 964)	6.4 (4.6)	5.5 (4.3)	7.4 (4.8)	7.6 (4.5)
Anxiety symptoms^3^ (score 0–21) (*n* = 965)	4.1 (3.6)	3.6 (3.3)	4.8 (3.8)	4.6 (3.7)
Fear of cancer recurrence^3^ (score 0–40) (*n* = 855)	11.9 (8.7)	11.3 (8.5)	13.1 (8.6)	11.7 (9.3)
Health literacy^4^ (score 12–48) (*n* = 821)	36.5 (5.3)	36.9 (5.2)	36.0 (5.3)	36.5 (5.5)
Lifestyle, *n* (%)				
Obese^5^ (*n* = 958)	168 (17)	86 (16)	49 (16)	33 (25)
Daily smoker (*n* = 967)	119 (12)	61 (12)	37 (12)	21 (16)
Physically inactive^6^ (*n* = 934)	520 (56)	270 (52)	170 (56)	80 (62)

*SD* standard deviation, *BCT* breast conserving treatment, *PA* physical activity

^1^Experiencing one or more of the following at least 3 times per week: difficulties falling asleep at night and/or waking up too early without being able to go back to sleep

^2^Increasing score implies better function

Increasing score implies worse symptoms

^4^Increasing score reflects better health literacy

^5^Defined as body mass index ≥ 30 kg/m^2^

^6^Defined as not meeting the public guidelines of at least 150 moderate-intensity physical activity per week. Numbers may not add up to 974 because of missing data. Percentages may not add up to 100 because of rounding

At diagnosis, 87% of the BCSs were in paid work and 8% held disability pension, while the remaining 5% held other work statuses (Table 2). At survey, 56% were in paid work, 34% received disability pension, 5% held early retirement, and 5% held other work statuses. All except one of the BCSs on disability pension at BC diagnosis still held disability pension at survey.

**Table 2:** Change in work status from diagnosis to survey among 974 Norwegian long-term breast cancer survivors

At diagnosis	At survey (~ 8 years post-diagnosis)			
	Paid work*, *n* = 547 (56%)	Disability pension,* n* = 330 (34%)	Early retirement,* n* = 48 (5%)	Other**, *n* = 49 (5%)
Paid work,* n* = 845 (87%)	530 (63%)	227 (27%)	48 (5%)	40 (4%)
Disability pension,* n* = 77 (8%)	1	76 (99%)	0	0
Other**, *n* = 52 (5%)	16 (31%)	27 (52%)	0	9 (17%)

^*^Paid work: working full-time, part-time, being self-employed, or on sick leave

^**^Other: work allowance, temporarily laid off, job seeker, homemaker, education, or military service

Of the 845 BCSs who were in paid work at diagnosis, work status was maintained in 63% and reduced in 37% (27% held disability pension, 6% retired early, and 4% held other work statuses) (Table 2). Of the BCSs who worked fulltime at diagnosis and maintained their work status (*n* = 434), 10% downgraded from full to part time work (data not shown).

Factors associated with reduced work status in univariable analyses are shown in Table 3. In the multivariable analyses, older age at diagnosis (OR 1.16, 95% CI 1.11–1.21), > 2 comorbid conditions (OR 2.27, 95% CI 1.16–4.32), lower cognitive function (OR 0.99, 95% CI 0.98–0.99), more fatigue (OR 1.02, 95% CI 1.01–1.03), and neuroticism (OR 1.57, 95% CI 1.00–2.46) were associated with increased risk of reduced work status. BCSs not living with children were less likely to reduce their work status than survivors living with children (OR 0.44, 95% CI 0.24–0.82). In addition, chemotherapy alone compared to no systemic treatment was significantly associated with reduced work status (OR 2.83, 95% CI 1.24–6.61).

**Table 3:** Factors associated with reduced work status (versus maintained work status) from diagnosis to survey among 845 Norwegian breast cancer survivors who were in paid work at diagnosis and within working age at survey

	Univariable analyses*		Multivariable analysis		
	OR	95% CI	OR	95% CI	*p*
Socio-demographic variables					
Living with partner					
Yes (reference)	1.0		1.0		
No	1.22	0.88–1.68	1.09	0.69–1.72	0.71
Living with children < 18 years					
Yes (reference)	**1.0**		1.0		
No	**1.56**	**1.09–2.24**	**0.44**	**0.24–0.82**	**0.01**
Years of education					
> 12 years (reference)	1.0		1.0		
≤ 12 years	1.18	0.89–1.57	0.91	0.61–1.37	0.66
Cancer-related variables					
Age at diagnosis	**1.07**	**1.04–1.09**	**1.16**	**1.11–1.21**	** < .001**
Systemic treatment burden					
No systemic treatment (reference)	1.0		1.0		
Chemotherapy alone	1.66	0.95–2.91	**2.83**	**1.24–6.61**	**0.01**
Endocrine therapy alone	**2.11**	1.11–4.0	1.32	0.56–3.08	0.52
Chemotherapy and endocrine therapy	1.20	0.76–1.89	1.12	0.59–2.13	0.73
Chemotherapy and trastuzumab	0.79	0.36–1.72	0.66	0.19–2.24	0.50
Chemotherapy, trastuzumab, endocrine therapy	1.49	0.87–2.55	1.51	0.70–3.28	0.30
Health variables					
Somatic comorbid conditions					
0 (reference)	**1.0**		1.0		
1–2	**2.26**	**1.57–3.26**	1.42	0.86–2.34	0.17
> 2	**4.75**	**3.01–7.50**	**2.27**	**1.16–4.32**	**0.02**
Sleep problems					
No (reference)	**1.0**		1.0		
Yes	**1.90**	**1.43–2.53**	1.41	0.93–2.14	0.10
Neuropathy					
Low (reference)	**1.0**		1.0		
High	**2.56**	**1.82–3.61**	1.41	0.86–2.32	0.17
Cognitive function	**0.98**	**0.97–0.99**	**0.99**	**0.98–.99**	**0.04**
Pain	**1.02**	**1.02–1.03**	1.0	0.99–1.01	0.99
Fatigue	**1.03**	**1.02–1.03**	**1.02**	**1.01–1.03**	** < .001**
Arm symptoms	**1.01**	**1.01–1.02**	1.01	0.99–1.02	0.23
Breast symptoms	**1.02**	**1.01–1.02**	0.99	0.99–1.01	0.68
Depressive symptoms	**1.09**	**1.06–1.13**	0.95	0.88–1.0	0.09
Fear of cancer recurrence	**1.03**	**1.01–1.04**	1.0	1.0–1.03	0.73
Neuroticism					
No (reference)	**1.0**		**1.0**		
Yes	**1.29**	**1.19–1.39**	**1.57**	**1.0–2.46**	**0.04**
Health literacy	**0.97**	**0.94–0.99**	0.99	0.96–1.03	0.82

Bold indicates statistically significant

^*^Only variables associated with reduced work status in univariable analyses with *p* < 0.25 are displayed in the table

### Self-perceived reasons for reduced work status

Among the 315 survivors with reduced work status, late effects after BC were reported as the main or partial reason for reduced work status in 64% (52% and 12%, respectively) of the BCSs. Among those with reduced work status receiving disability pension (*n* = 227), 83% responded that BC was the reason for disability.

### Attrition analysis

Compared to responders in the SWEET study in total, non-responders (*n* = 1448) were significantly older (53.2 years at diagnosis), had lower Ki67 values (mean 27 versus 31), and a larger proportion was HER-2 negative (85% versus 81%), while there were no differences in tumor size, nodal status, hormone receptor status, or type of surgery. Characteristics of responders to the SWEET study in total and those aged ≥ 67 years at survey are displayed in Supplementary file.

## Discussion

In this nationwide study of long-term BCSs below retirement age, the majority of those who were employed at diagnosis maintained their work status 8 years later (63%). Living with children, older age at diagnosis, having received chemotherapy (vs no systemic treatment), > 2 comorbid conditions, more fatigue, lower cognitive function, and high neuroticism increased the risk of BCSs reducing their work status long-term. The BC diagnosis in itself and late effects were the main or partial self-perceived reasons for reduced work status and disability.

Previous research has clearly demonstrated that most BCSs who are employed at diagnosis, return to their work within 2 years post-treatment [9, 11]. The extent to which long-term BCSs remain in the paid work force is less known [11]. In a register-based study, Paalman et al. found that close to 60% of Dutch BCSs treated between 2000 and 2005 were employed 7 years later [15]. In a cross-sectional study of Israeli BCSs (*n* = 206), 67% were still employed 8 years after diagnosis [13], and in a German cross-sectional study including 135 BCSs, 70% had returned to work with full or reduced working time 5 years after surgery [14]. In another recent cross-sectional study, Peipins et al. found that over 80% of about 1600 US BCSs sustained their employment on average 5 years after diagnosis [12]. Although these employment rates among long-term BCSs are not directly comparable to findings in our study due to different socioeconomic and healthcare systems, they all indicate that the majority of BCSs maintain their work status also beyond the first years post-diagnosis.

Our findings that 87% of the BCSs were in paid work and 8% received disability pension at BC diagnosis (mean age 48.1 years) correspond well with national work status statistics. Among Norwegian women aged 40–54 years in 2012, about 82% were in paid work and 10% received disability pension [37, 38]. In 2019, this statistics showed that 64% of women aged 55–66 years were in paid work and 23% received disability pension, while the corresponding numbers among the BCSs at survey were 56% and 34% [37, 38]. Our findings therefore indicate an increased risk of unemployment and disability pension among long-term BCSs compared to women in the general population, also after treatment in the more modern era.

Socio-demographic aspects and personality as well as health variables, late effects, and treatment modalities were significantly associated with reduced work status 8 years after the diagnosis. Several of these factors are consistent with prior findings on adverse work outcomes among BCSs mainly within 2 years from diagnosis [6, 9]. In a systematic review, older age at diagnosis, chemotherapy, multiple comorbid diseases, subjective cognitive dysfunction, and fatigue were associated with a reduced risk of returning to work during the first years after BC [9]. Another systematic review on functional impairments and work-related outcomes in BCSs reported no association between cognitive dysfunction diagnosed with neuro-psychological tests and work-related outcomes, whereas the results of studies using self-reported measures were ambiguous [6].

Importantly, a concern with neuro-psychological tests is that they do not take into account the environment at the workplace (i.e., lacks ecological validity), and therefore they may not reflect the actual cognitive challenges BCSs face when returning to work [6]. In qualitative studies, BCSs report cognitive impairments and fatigue as main barriers of returning to and managing work [3, 6]. Although cognitive impairments and fatigue usually diminish shortly after treatment completion [39], some BCSs may experience these symptoms for up to 10 years [40, 41]. In accordance with this, the BCSs in our sample had considerably lower cognitive function score (71.4 vs 86.6) and higher symptom score of fatigue (40.8 vs 29.1) than previously reported among Norwegian women from the general population aged 50 to 59 years [42].

The BCSs reported late effects as one of the main reasons for reduced work status and disability. To promote a sustainable work life for long-term BCSs, health personnel, employers, and labor and welfare administration personnel should be aware that late effects such as cognitive dysfunction and fatigue may persist for years after diagnosis and contribute to reduced work participation. BCSs who are 5–10 years from diagnosis report that flexibility from the employer and work place accommodations are highly beneficial in order to remain within the work force [3]. For example, for BCSs with fatigue, practical help, adjustments in tasks at work, and flexibility in work hours may facilitate work sustainability [3].

Physical activity was not associated with work status in this study, contrasting prior findings showing a positive association between regular exercise and return to work among BCSs examined few years after diagnosis [18]. Modifiable factors that are previously described as associated with difficulty returning to work after BC, including reduced physical functioning, arm problems, and fatigue [4, 6], can be improved by physical exercise [43–45]. Therefore, we consider physical activity an important component of multidisciplinary rehabilitation programs aiming to increase work participation among long-term BCSs.

Neuroticism is a basic personality trait, defined as a tendency to react with negative emotions when faced with stress [46] and may indeed influence coping and several other factors in relation to work. In BCSs, neuroticism is associated with several negative health outcomes, such as depression, anxiety, and reduced quality of life [19]. Further, previous research has shown that optimism, the ability to adapt to new circumstances, and to take advantage of emerging opportunities are crucial for return to work and a sustainable work life after BC [47]. Our results indicate that in the clinical setting, BCSs with high neuroticism may require more support, information, and encouragement in order to return to and remain in paid work after diagnosis.

### Strengths and limitations

This study is one of few evaluating work status among BC survivors beyond the 5 first years after diagnosis. A major strength is the large nationwide sample of long-term survivors diagnosed with stage I-III BC in 2011 or 2012 within working age in Norway. The sample was extracted by the CRN, ensuring a high degree of completeness and accuracy as reporting to the CRN is required by law [48]. Another strength is the inclusion of several patient-reported outcomes as explanatory variables, assessed by established instruments with satisfactory psychometric properties. Furthermore, we consider it a strength that the BCSs were asked to report if the inverse work outcomes were caused by the BC and late effects.

This is a cross-sectional study, and consequently we present associations between variables and make no inferences on causality. Another limitation is the lack of information on the work status trajectory between diagnosis and survey, whether the BCSs changed their occupation or work tasks, and information about other major life events during the eight past years which may have influenced work status. This limitation is particularly relevant regarding the women who were excluded because they had reached retirement age at survey, and information about whether they returned to work in the years between diagnosis and retirement was lacking. Future studies should explore the work trajectory among BCSs who are only a few years from retirement age when diagnosed with BC. Furthermore, job resources and social support at the workplace were not assessed, which also represent an important area for future studies within this population. The response rate of 49% is in accordance with the expected response rates for population-based health surveys (40–50%) [49]. The participants in our study differed only slightly from the invited total cohort being somewhat younger at diagnosis and with more aggressive tumor characteristics. Although we cannot exclude that the study participants were more heavily treated than non-responders, we believe our results are representative for BCSs still in working age. Our study reflects the Norwegian welfare system in which sick leave compensation and disability pension are mostly financed through the government by taxation. Our results may not be generalizable to countries with different and less comprehensive welfare systems than Norway.

## Conclusion

Two thirds of BCSs within working age who were in paid work at diagnosis were also working 8 years later. Reduced work status was associated with older age, living with children, > 2 comorbid conditions, chemotherapy, fatigue, cognitive dysfunction, and neuroticism. Identifying and improving fatigue and cognitive dysfunction early in BC survivorship are important to secure return to work after BC, needs to be acknowledged by the clinical community and employers, and should be included in future interventions to promote work sustainability in long-term BCSs.

### Supplementary Information

Below is the link to the electronic supplementary material.

Supplementary file1 (DOCX 20.6 KB)

## Data Availability

All data is available at the National Advisory Unit for Late Effects after Cancer Treatment, Department of Oncology, Oslo University Hospital, the Radium Hospital, Oslo, Norway.
