# Supplementary material for: Work status changes and associated factors in a nationwide sample of Norwegian long-term breast cancer survivors
Source: J Cancer Surviv. 2022 Mar 22;18(2):375–84. doi: 10.1007/s11764-022-01202-2 (PMC10960762; doi:10.1007/s11764-022-01202-2)
Supplement: Supplementary file 1 — Supplementary file1 (DOCX 20.6 KB) [file 11764_2022_1202_MOESM1_ESM.docx]

**Table 4:** characteristics of the total responding breast cancer survivors (BCSs) and BCSs aged ≥ 67 years at survey

|  | Total sample | BCSs aged ≥ 67 years at survey |
| --- | --- | --- |
|  | N=1355 | N=349 |
| **Socio-demographic variables** |  |  |
| Age at survey, mean (SD) | 59.9 (8.7) | 70.0 (2.0) |
| Living with partner, n (%) | 994 (74) | 235 (67) |
| Living with children < 18 years, n (%) | 202 (15) | 5 (1) |
| Years of education, n (%) |  |  |
| Long (>12 years) | 691 (51) | 131 (38) |
| Short (≤ 12 years) | 647 (48) | 210 (62) |
| *Missing* | *17 (1)* | *8 (2)* |
| **Cancer-related variables** |  |  |
| Mean age at diagnosis (SD) | 51.9 (8.6) | 62.0 (2.0) |
| Mean years since diagnosis (SD) | 7.5 (0.6) | 8.1 (0.7) |
| Stage, n (%) |  |  |
| I | 606 (45) | 206 (59) |
| II | 486 (36) | 102 (29) |
| III | 108 (8) | 20 (6) |
| *Missing* | *155 (12)* | *21 (6)* |
| Treatment, n (%) |  |  |
| BCT | 796 (59) | 249 (71) |
| Mastectomy | 559 (41) | 100 (29) |
| Radiotherapy | 1087 (80) | 289 (83) |
| *Systemic treamtent* |  |  |
| No systemic treatment | 245 (18) | 108 (31) |
| Chemotherapy alone | 162 (12) | 39 (11) |
| Endocrine treatment alone | 172 (13) | 87 (25) |
| Chemotherapy+endocrine therapy | 534 (39) | 77 (22) |
| Chemotherapy+ trastuzumab | 64 (5) | 11 (3) |
| Chemotherapy+ trastuzumab +endocrine therapy | 166 (12) | 23 (7) |
| *Missing* | *12 (1)* | *4 (1)* |
| **Health variables, n (%)** |  |  |
| Somatic comorbid conditions |  |  |
| 0 | 290 (21) | 49 (14) |
| 1-2 | 731 (54) | 173 (50) |
| >2 | 326 (24) | 125 (36) |
| *Missing* | *8 (0.6)* | *2 (0.6)* |
| Sleep problems^1^ | 592 (44) | 129 (38) |
| *Missing* | *26 (2)* | *11 (3)* |
| Neuropathy | 277 (20) | 70 (20) |
| *Missing* | *33 (2)* | *18 (5)* |
| High neuroticism | 526 (39) | 124 (36) |
| *Missing* | *24 (2)* | *8 (2)* |
| **Health variables, mean (SD)** |  |  |
| *EORTC-QLQ-C30/BR-23 (score 0-100)* |  |  |
| Cognitive function^2^ | 74.3 (24.9) | 82.7 (20.7) |
| Pain^3^ | 28.1 (29.4) | 26.5 (29.1) |
| Fatigue^3^ | 37.8 (27.6) | 29.9 (25.5) |
| Arm symptoms^3^ | 20.7 (24.6) | 16.6 (23.2) |
| Breast symptoms^3^ | 16.2 (19.3) | 14.0 (18.2) |
| Depressive symptoms^3^ (score 0-27) | 5.9 (4.5) | 4.5 (4.1) |
| Anxiety symptoms^3^ (score 0-21) | 3.8 (3.6) | 3.0 (3.5) |
| Fear of cancer recurrence^3^ (score 0-40) | 11.7 (8.7) | 10.9 (8.9) |
| Health literacy^4^ (score 12-48) | 36.2 (5.3) | 35.2 (5.3) |
| **Lifestyle, n (%)** |  |  |
| Obese^5^ | 239 (18) | 62 (18) |
| *Missing* | *25 (2)* | *7 (2)* |
| Daily smoker | 172 (13) | 51 (15) |
| *Missing* | *12 (1)* | *4 (1)* |
| Physically inactive^6^ | 717 (53) | 179 (51) |
| *Missing* | *106 (8)* | *57 (16)* |

SD= standard deviation, BCT= breast conserving treatment, PA: physical activity.^1^ experiencing one or more of the following at least 3 times per week; difficulties falling asleep at night and/or waking up too early without being able to go back to sleep ^2^ Increasing score implies better function, ^3^ Increasing score implies worse symptoms, ^4^ increasing score reflects better health literacy, ^5^defined as body mass index ≥30 kg/m^2^, ^6^defined as not meeting the public guidelines of at least 150 moderate-intensity physical activity per week. Numbers may not add up to 1355 because of missing data. Percentages may not add up to 100 because of rounding.
